# Novel coil design and analysis for high-power wireless power transfer with enhanced *Q*-factor

**DOI:** 10.1038/s41598-023-31389-y

**Published:** 2023-03-14

**Authors:** Charles Marfo Awuah, Patrick Danuor, Jung-Ick Moon, Young-Bae Jung

**Affiliations:** 1 Department of Electronic Engineering, Hanbat National University, Daejeon, 34158 South Korea; 2 Radio and Satellite Research Division, Electronics and Telecommunications Research Institute (ETRI), Daejeon, South Korea

**Keywords:** Electrical and electronic engineering, Power stations

## Abstract

The power transfer efficiency (PTE) is a crucial aspect for effective wireless power transfer (WPT) applications. The quality factor (*Q*) of the WPT coil plays a critical role in ensuring higher PTE. In this paper, a novel method of improving the *Q* of a WPT coil is proposed. Resistance reduction techniques are presented which involves variation of the trace pitch, width, and thickness. This approach targets the high AC losses centered in the inner turns, which subsequently results in an increased *Q*. Numerical analysis with respect to the inductance and resistance models are presented, analyzed, and compared to that of the EM simulation results. To verify the efficacy of the proposed coil structure, a prototype is fabricated where good agreement is achieved between the measured and simulated results. The proposed coil attained a quality factor increment of about 19.24% at 85 kHz in comparison to the conventional one. The proposed technique can be used to optimize planar spiral coils to attain higher *Q*.

## Introduction

With the growing demand on remote transfer of power, there has been a significant amount of research into wireless power transfer (WPT) technology^1^. The effective use of WPT systems pivots on convenience for the user where an electronic device could be utilized without plugging to a power source^1,2^. Moreover, WPT systems have expanded to include applications such as electric vehicle charging, smart watches, implantable microelectronic devices (IMDs) and the Internet of things (IoT)^3^.

The design of highly efficient coils remains a crucial aspect for effective WPT operation^4^. However, maximizing the efficiency of WPT systems becomes complex due to the existence of a trade-off behaviour in optimizing the coil parameters for power transfer efficiency (PTE), which suggests that optimizing one parameter makes the other less effective^5^. This becomes a challenge where for instance, small solenoid coils for biomedical implants need to be realized with lightweight and miniaturized coils yet maintaining high efficiency^6^.

Power transfer by WPT systems can be influenced by the quality factor (*Q*) in the sense that the transmit (TX) and receive coil (RX) with a high *Q* can result in high efficiency of WPT systems. For planar coils, the *Q* mainly depends on the inductance and the resistance of the coil. Thus, the high *Q* indicator becomes crucial in coil design since losses are intensely caused by the high-frequency winding resistance^7–9^.

A number of literature studies have been done with respect to maximizing the PTE of WPT systems by improving the *Q* of the coil. A divide-and-merge technique to reduce skin effect and improve the *Q*-factor of thin film printed coils is proposed in^10^. However, this technique is complex in its implementation and only applicable to spiral winding with just a single coil turn.

Recently, a number of coil layout optimization strategies have been proposed pertaining to the coil width, coil pitch and trace thickness^11–16^. Lopez-Villegas et al.^11^ for example, introduced a layout optimization strategies which involves the varying coil trace width to reduce the overall resistance of the coil windings are proposed based on analytical model with investigations on inductor coil series resistance (i.e., ohmic and induced losses) due to conduction and eddy currents, respectively. A method of improving the performance of printed planar coils through the varying of trace width and turn-to-turn spacing (pitch) has been presented in^12^. Moreover, the *Q* of a hollow spiral winding is improved by the combination of non-uniform coil turn width strategy with alteration of the internal diameter or the total number of coil turns altogether in^13,14^. Thus, the losses in the inner turns of planar windings are suppressed which subsequently reduces the resistance of the coil.

Furthermore, a design layout algorithm to minimize the resistance of coil winding with variable coil turn width is presented in^15^. Again, the variations in coil turn width to achieve higher *Q*-factor by adjusting the gaps between coil turns, scaling factor and the number of turns has been demonstrated by Kim et al.^16^.

In this paper, a novel and optimized coil layout structure is proposed which utilizes the combination of the coil trace pitch, width, and thickness variation method to maximize the *Q* of the coil. This combined technique reduces the high AC losses of the coil turns concentrated in the innermost part of the spiral planar coil. Besides, the resistance of the coil is reduced without compromising on its inductance since no coil turn is removed. Additionally, the dimension of the proposed coil is maintained compared to that of the conventional one. The experimental results reveal a *Q*-factor increment of 19.24% at 85 kHz of the proposed coil compared to that of the conventional one which signifies the feasibility of the proposed coil for miniaturized and high performance WPT applications.

Following the introduction, the *Q*-factor modelling for the proposed coil is given in "*Q*-factor modelling" section. "Parametric study" section presents parametric studies of the effect of the trace pitch, thickness, and width on the resistance of the coil. The analysis of the conventional a proposed coil structure is presented in "Analysis of the conventional and proposed rectangular coil structure" section. "Experimental results and verification" section presents the experimental results of the proposed coil while the conclusions are drawn in “Conclusion” section.

## *Q*-factor modelling

### Modelling of inductance (L)

**Figure 1:**
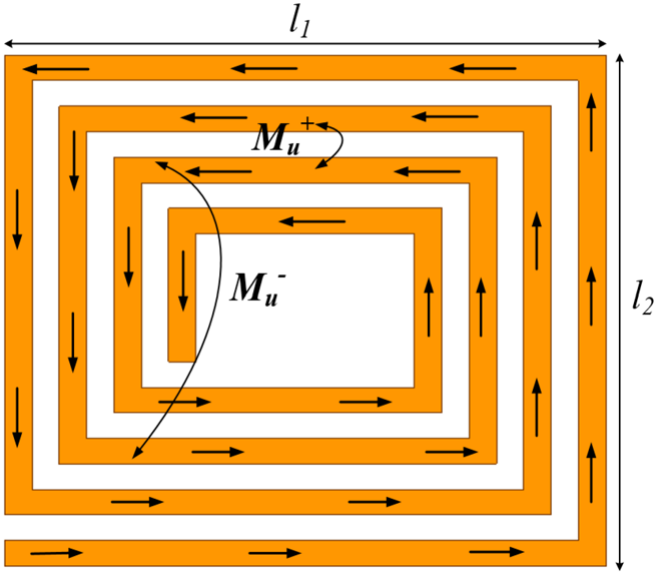
2D layout of a multi-turn rectangular planar coil^18^.

The total inductance of a multi-turn rectangular planar coil as shown in Fig. 1 can be deduced by the addition of the self-inductance (*L*_*s*_) of each coil turn, the positive mutual inductance (*M*_*u*_^+^) and the negative mutual inductance (*M*_*u*_^*−*^) between each coil turn as in^17,18^.

1 $$L_{T} = L_{{_{S} }} + [M_{{u^{ + } }} - M_{{u^{ - } }} ]$$

The self-inductance (*L*_s_) of a rectangular coil with *N* turns can be expressed as

2 $$L_{s} = \sum\limits_{i = 1}^{4n} {\frac{{\mu_{o} }}{6.2832}l_{n} \left[ {In\left( {\frac{{2_{n} }}{{w_{n} + t_{n} }}} \right) + 0.5 + \frac{{w_{n} + t_{n} }}{{3l_{n} }}} \right]}$$

where *n*,$\mu_{o}$,$w_{n}$ and $t_{n}$ denotes the coil trace number from the outer section to the inner section of the coil, permeability of free-space of value 4π × 10^–7^ H/m, width and thickness of the *n*th coil turn, respectively. The length of one coil turn (*l*_*n*_) can also be expressed as^19^

3 $$l_{n} = \left( {\frac{n}{2} - 1} \right)(w_{n} + p_{n} )\frac{{x_{n} }}{2}$$

where,

4 $$x_{n} = l_{1} + l_{2} + ( - 1)^{n} (l_{2} - l_{1} )$$

The mutual inductances (i.e., positive, and negative) between two parallel neighbouring coil turns with lengths *li* and *lj* can be calculated as given in^20^. The positive mutual inductance, *M*_*u*+_ and the distance (*D*_max_) between the coil turns is given as

5 $$M_{{u^{ + } }} = 2\sum\limits_{i = 1}^{n} {\sum\limits_{j = i + 1}^{n} {\sum\limits_{k = 1}^{4} {M_{4} } } } (i - 1) + k,4(j - 1) + k$$

6 $$D_{\max } = (j - i)(w_{n} + p_{n} )$$

where *i, j* represents the turn number (i.e., from outermost to innermost turn). $k$ denotes the trace number of that turn. In addition, the negative mutual inductance, and the distance (*D*_min_) between coil turns in that regard is also given as in Eqs. (7) and (8) below^19^

7 $$M_{{u^{ - } }} = 2\sum\limits_{i = 1}^{n} {\sum\limits_{j = 1}^{n} {\sum\limits_{k = 1}^{2} {M_{4} } } } (i - 1) + k,4(j - 1) + k + 2$$

8 $$D_{\min } = \frac{{x_{n} }}{2}(w_{n} + p_{n} )(i + j - 2)$$

### Modelling of resistance (R_total_)

For the resistance of the planar spiral coil, we consider two components i.e., the resistances caused by the skin-effect and the resistance due to the current crowding (i.e., proximity-effect) between coil turns.

#### Skin-effect resistance (R_skin_)

The resistance due to skin-effect emerges from the current in the conductor gathering at the edges. Due to coil turn thickness, pitch and width variation in the proposed coil layout, the skin effect resistance from can be expressed as^12,21^

9 $$R_{skin} = \sum\limits_{n = 1}^{N} {\frac{{l_{n} }}{{\sigma w_{n} \alpha^{\frac{1}{2}} \beta }} \times \frac{1}{\tau }}$$

where $\sigma$ represents the conductivity of the coil. The parameters $\sigma$, $\beta$ and $\tau$ can be represented in Eq. (10) as

10 $$\left\{ {\begin{array}{*{20}c} {\alpha = \left( {\frac{1}{{\pi \sigma \mu_{o} f}}} \right)} \\ {\beta = (1 - e^{{ - \frac{{t_{n} }}{{p_{n} }}}} )} \\ {\tau = (1 + \frac{{t_{n} }}{{w_{n} }})} \\ \end{array} } \right.$$

#### Proximity-effect resistance (R_prox_)

Resistance due to the nearness of neighbouring conductors or current crowding impact is caused by the currents induced by the magnetic fields from other coil turns. The proximity-effect resistance (*R*_*prox*_) can be expressed as^20,22^.

11 $$R_{prox} = \sum\limits_{n = 1}^{N} {(\frac{4\pi }{\sigma }l_{n} \Phi_{n} H_{n}^{2} } )$$

where,

12 $$\left\{ {\Phi_{n} = - 0.998 + 0.112\frac{{w_{n} }}{{t_{n} }} - 0.689\frac{{t_{n} }}{\delta } + 0.543\frac{{w_{n} }}{\sigma } + 0.002\left( {\frac{{w_{n} }}{{t_{n} }}} \right)^{2} } \right.$$

$H_{n}$ represents the magnetic field perpendicular to the surface of the turn under the current excitation of 1A. The total resistance (*R*_*total*_) of the spiral planar coil can therefore be expressed as the sum of the skin-effect and proximity-effect resistances as in Eq. (13)

13 $$R_{total} = R_{skin} + R_{prox}$$

### Quality factor modelling

The modelling of the inductance and resistance equations above highlights the impact of the coil trace thickness and width variations of the rectangular planar coil. Therefore, the *Q*-factor modelling is based on the total inductance and resistance in Eqs. (1) and (13), respectively as

14 $$Q = \frac{{\omega L_{T} }}{{R_{total} }}$$

## Parametric study

To study the effect of varying the coil parameters (i.e., trace thickness, width, and pitch) on the resistance of the conventional spiral planar coil, parametric studies are presented in this section. The numerical analysis using MATLAB^23^ as well as the electromagnetic (EM) simulation results using ANSYS Maxwell Electromagnetics software^24^ are both presented and compared.

### Parametric analysis with trace width variation

**Figure 2:**
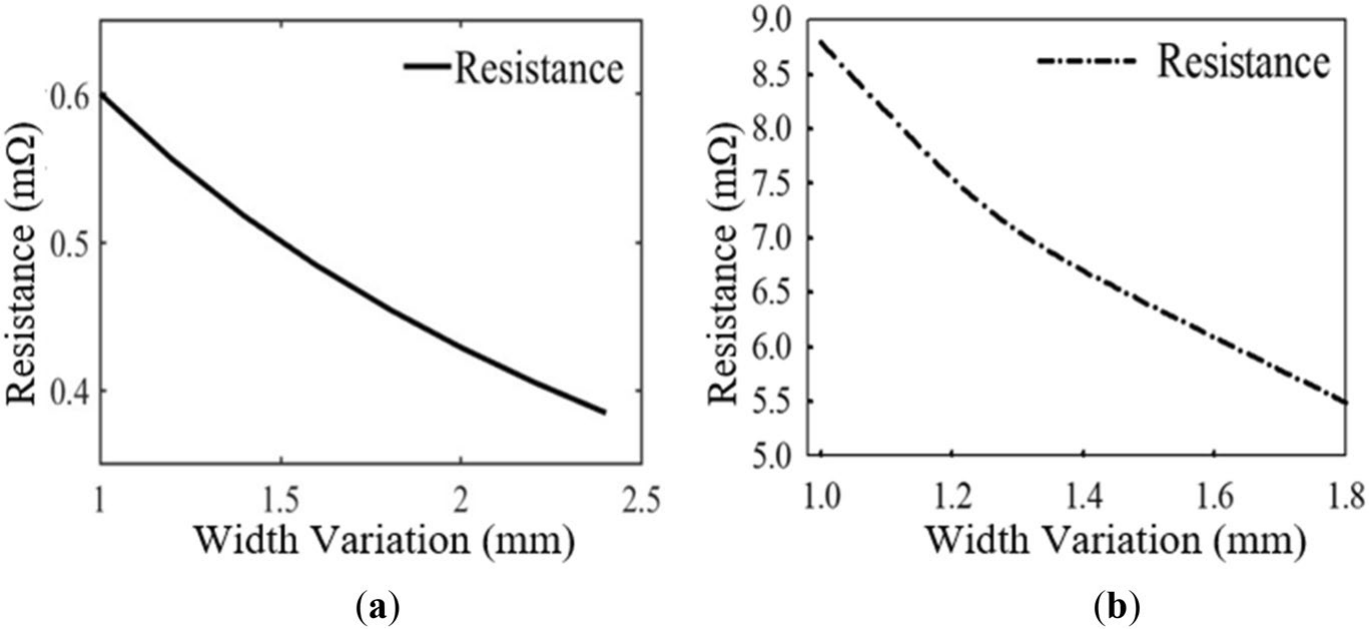
Coil resistance variation with changing trace width (**a**) numerical method and (**b**) EM simulation.

To analyse the influence of varying the trace width on the coil resistance, the thickness and pitch of the coil are kept constant with values of 2.4 mm and 1.1 mm, respectively. Coil turn widths ranging between 1 and 1.8 mm (i.e., 1, 1.2, 1.4 and 1.8 mm) are selected for the parametric studies. Figure 2a,b show the variation of the coil resistance with increasing coil turn width for the numerical and EM simulation results, respectively. The numerical method based on Eq. (9) is utilized to compute the coil resistance. Table 1 summarizes the EM simulation results.

**Table 1:** Simulation results of the conventional coil structure with different widths (*w*).

Coil structure	*L* (μH)	*R* (mΩ)	*Q*
Coil *1* (*w* = 1.0 mm)	2.89	8.78	175.79
Coil *2* (*w* = 1.2 mm)	2.92	7.55	206.55
Coil *3* (*w* = 1.4 mm)	2.95	6.70	235.15
Coil *4* (*w* = 1.8 mm)	3.03	5.49	294.76

It can be realized from Table 1 that as the width of the coil turn increases, the resistance of the coil reduces. Thus, there is an inverse relationship between the coil turn width and the resistance as exhibited in Fig. 2a,b. This happens because, as the coil turn width increases, the cross-sectional area of the conductor for the coil winding also increases thereby reducing the resistance of the coil. Generally, as the cross-sectional area of a conductor increases, its resistance decreases. This can be inferred from Eq. (15), where $\rho ,l,$ and $A$ is the resistivity, length, and cross-sectional area of the conductor, respectively.

15 $$R = \rho \frac{l}{A}$$

### Parametric analysis with trace thickness variation

**Figure 3:**
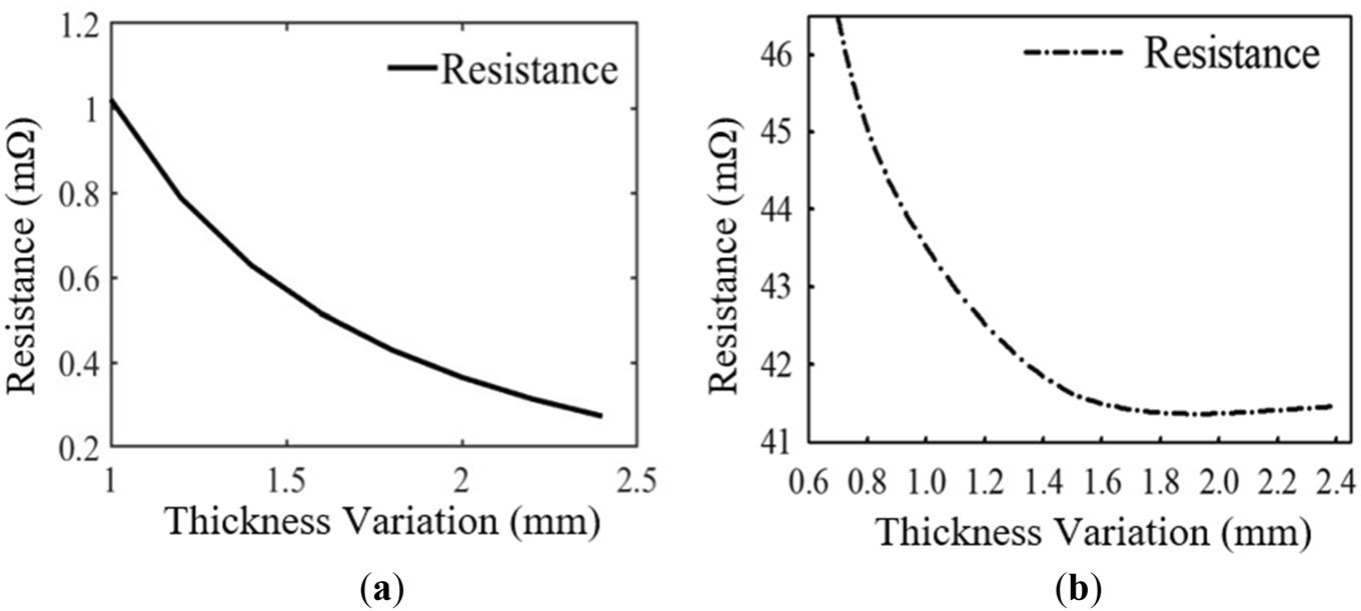
Coil resistance variation with changing trace thickness (**a**) numerical method and (**b**) EM simulation.

In order to evaluate the behaviour of the coil resistance in varying the coil turn thickness while keeping the width and pitch of the coil constant, values ranging from 0.7 to 2.4 mm are selected (i.e., 0.7, 0.9, 1.5 and 2.4 mm). A plot of the coil resistance with respect to coil thickness variation is shown in Fig. 3a,b (i.e., EM simulation and numerical results, respectively). Based on the EM simulations, the summary results of coil thickness variations are given in Table 2.

**Table 2:** Simulation results of the conventional coil structure with different thickness (*t*).

Coil structure	*L* (μH)	*R* (mΩ)	*Q*
Coil *I* (*t* = 0.7 mm)	3.12	50.73	32.87
Coil *II* (*t* = 0.9 mm)	3.06	44.17	37.00
Coil *III* (*t* = 1.5 mm)	2.96	41.62	37.98
Coil *IV* (*t* = 2.4 mm)	2.82	41.46	36.33

From Table 2 it is realized that, as the thickness of the coil increases, the resistance in contrast decreases. Thus, increasing the thickness of the coil windings, in effect increases the cross-sectional area of the conductors. From Eq. (15), it can be deduced that the cross-sectional area, *A* is inversely proportional to the resistance of a coil windings. Therefore, as the thickness of the coil turn increases, the resistance of the coil decreases.

The discrepancies between the EM simulation and numerical results of Figs. 2 and 3 is due to the reason that the EM simulation takes into consideration both Eqs. (9) and (11) but the numerical simulation considers only Eq. (9). This is because, it is difficult to model the parameter *H*_*n*_ (i.e., the magnetic field perpendicular to the surface of the *n*_*th*_ turn) in the numerical computation of the proximity-effect resistance in Eq. (11). Notwithstanding, both the EM and numerical results show a similar behaviour in terms of the trace width and thickness variation with changing widths and thicknesses, respectively.

### Parametric analysis with coil pitch variation

**Figure 4:**
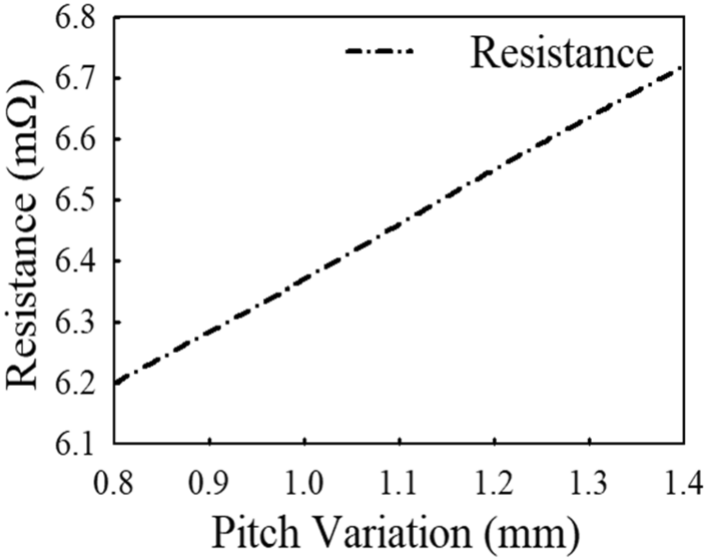
EM simulated results of the coil resistance variation with changing coil pitch.

In varying the coil pitch to examine the behaviour of the coil resistance, the coil’s width and thickness are kept constant. In the analysis, spacing values ranging between 0.8 and 1.4 mm (i.e., 0.8, 1.0, 1.2 and 1.4 mm) are selected for the studies. Figure 4 represents the graph of coil resistance with respect to coil pitch variations. Table 3 summarizes the EM simulation results of the coil’s resistance with changing coil pitch.

**Table 3:** Simulation results of the conventional coil structure with different pitches (*p*).

Coil structure	*L* (μH)	*R* (mΩ)	*Q*
Coil *A* (*p* = 0.8 mm)	3.04	6.20	33.16
Coil *B* (*p* = 1.0 mm)	3.05	6.37	37.52
Coil *C* (*p* = 1.2 mm)	3.08	6.55	36.32
Coil *D* (*p* = 1.4 mm)	3.10	6.72	34.86

It is realized from Table 3 that as the spacing between coil turns increases, the resistance of the coil increases. Thus, increasing the spacing of between neighbouring coil turns stretches the coil over a longer length (*l*) which increases the resistance of the conductor as expressed in Eq. (15). Thus, widening the spacing between coil turns results in magnetic field leaking out of the coil and creating more opposition to magnetic field linking other neighbouring coil turns which increases resistance of the coil^12,20^.

Owing to magnetic fields seeping out of the coil turns and producing additional resistance to the magnetic fields connecting other nearby coil turns, varying of the pitch of a coil has an effect particularly on *R*_*prox*_^12^. But the inclusion of *H*_*n*_ in Eq. (11) makes it difficult to compute values in predicting the behaviour of resistance with pitch variation analytically. In view of that, only the EM simulation results are presented to show the resistance behaviour with varying pitch.

## Analysis of the conventional and proposed rectangular coil structure

### Conventional coil

**Figure 5:**
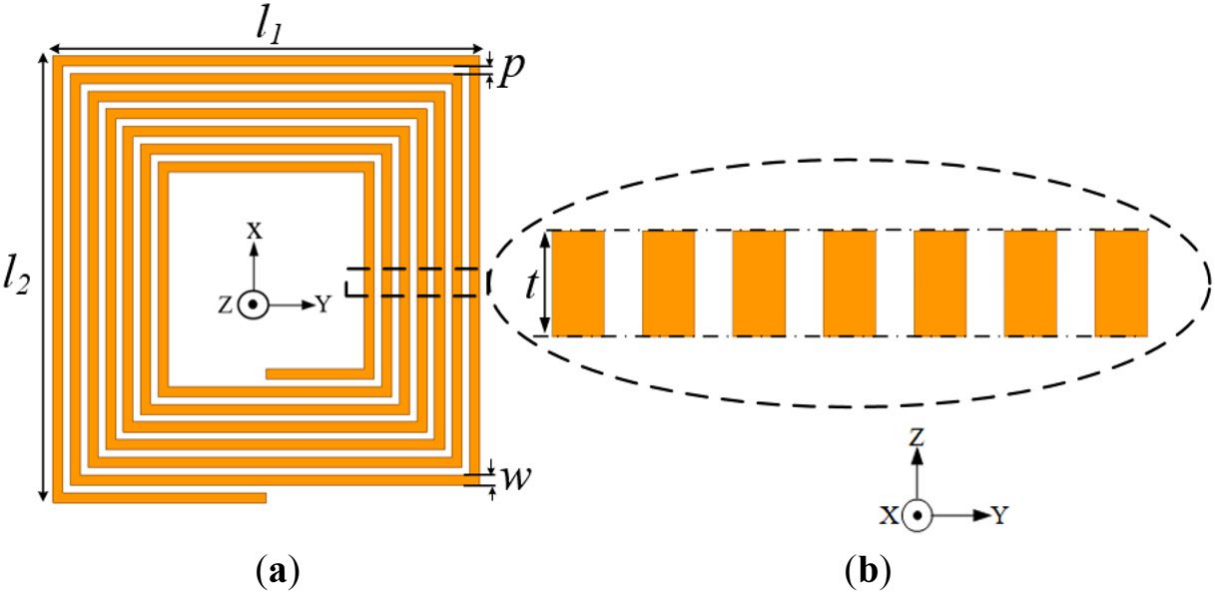
Conventional coil geometry (**a**) isometric view and (**b**) cross section of the exploded view of each coil turn with same thickness.

Figure 5a provides an isometric view of the conventional rectangular planar coil structure, where *N*, *p, w,* and* t* denote the number of coil turns, pitch, width and thickness of coil turns, respectively. The conventional coil is made up of seven (7) turns with a constant spacing between coil turns (*p*) of 1.1 mm, uniform turn thickness (*t*) of 2.4 mm and uniform turn width (*w*) of 1.5 mm as illustrated in Fig. 5b.

**Figure 6:**
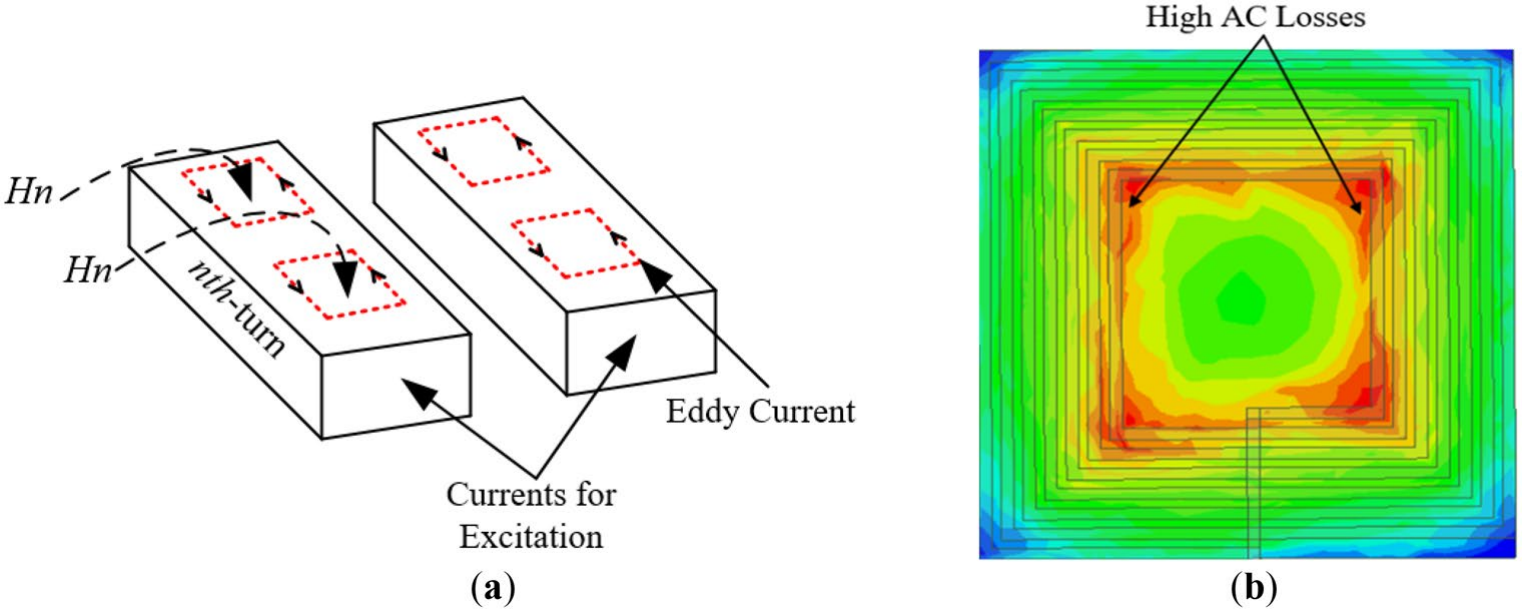
Illustration of (**a**) proximity-effect formation (**b**) magnetic field intensity (*H*_n_) of the rectangular planar coil winding^24^.

*Q*-Factor enhancement of the coil can be achieved when there is resistance reduction and little or no significant alteration in the inductance of the coil^12^. This is because, resistance is inversely proportional to *Q* from Eq. (14). Skin-effect resistance (*R*_*skin*_) and proximity-effect resistance (*R*_*prox*_) makes up the total resistance (*R*_*total*_) in the planar coil^12,25^. The former takes place when AC current supplied into the conductor gathers at its edges, increasing its resistance, whiles the latter happens when the magnetic fields from the current of nearby conductors generate eddy current in the *nth* turn of the conductor^20^ as illustrated in Fig. 6a.

The production of magnetic fields by AC currents are found inside and outside of the conductors existing within the neighbouring conductors. High losses in planar winding are due to the fact that, they endure very high intensity of magnetic field perpendicular to their surfaces creating high-frequency eddy current losses. For a coil of multiple turns, these losses are concentrated within the inner conductor, i.e., close to the middle section and gradually decreases in the outer section of the coil where the magnetic field intensity is highest^13,26^.

It can be realized from^13^ that accumulated magnetic fields in the inner turns of the coil increases the eddy-current losses located in the coil inner section as shown in Fig. 6b.

Again, due to the direct proportionality between proximity effect resistance (*R*_*prox*_) and magnetic field intensity (*H*_*n*_)^19^, the intensity of the proximity-effect resistance increases with increasing magnetic field. From the simulation results, an inductance and resistance of 2.82 μH and 41.46 mΩ, respectively was achieved at the operating frequency of 85 kHz.

### Conventional coil with unequal pitches

One way of reducing the total resistance of the planar coil is to implement pitch variation i.e., employing different spacing between each of the coil turns. The proximity impact of neighbouring coil turns due to currents induced by the *H*-fields from other conductors, increases the proximity-effect resistance (*R*_*prox.*_)^12^.

**Figure 7:**
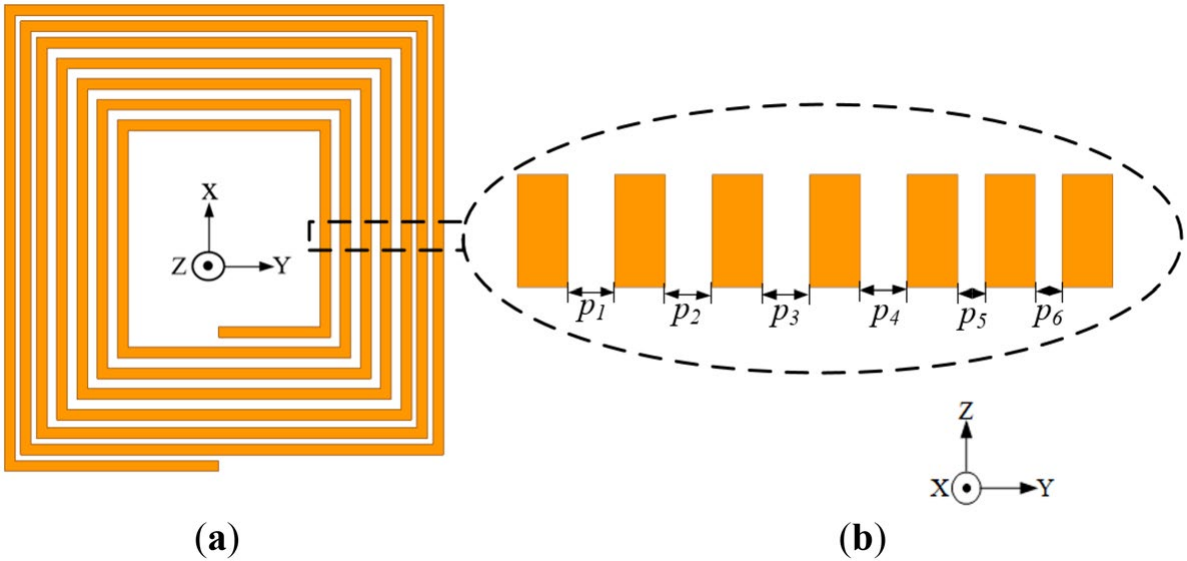
Configuration of the conventional coil structure with wider spacing between inner coil turns relative to the outer turns (Coil-*P*) (**a**) isometric view and (**b**) cross section of the exploded view of each coil turn with different pitches.

The geometry of the conventional coil with varying pitches incorporated between each coil turn (labelled as Coil-*P* for simplicity) is presented in Fig. 7. The coil is designed with the same parameters as that of the conventional coil presented in "Conventional coil" section, however with different pitches (i.e., *p*_*1*_, *p*_*2*_, *p*_*3*_, *p*_*4*_, *p*_*5*_ and *p*_*6*_ of 1.4, 1.4, 1.4, 1.4, 0.8 and 0.8 mm, respectively). The spacing between the coil turns in the inner section of the coil are widened compared to the outermost coil turns. This is because the intensity of magnetic fields produced by the AC currents is highest in the innermost part of the rectangular planar coil resulting to high AC losses, thereby increasing the resistance of the coil.

**Figure 8:**
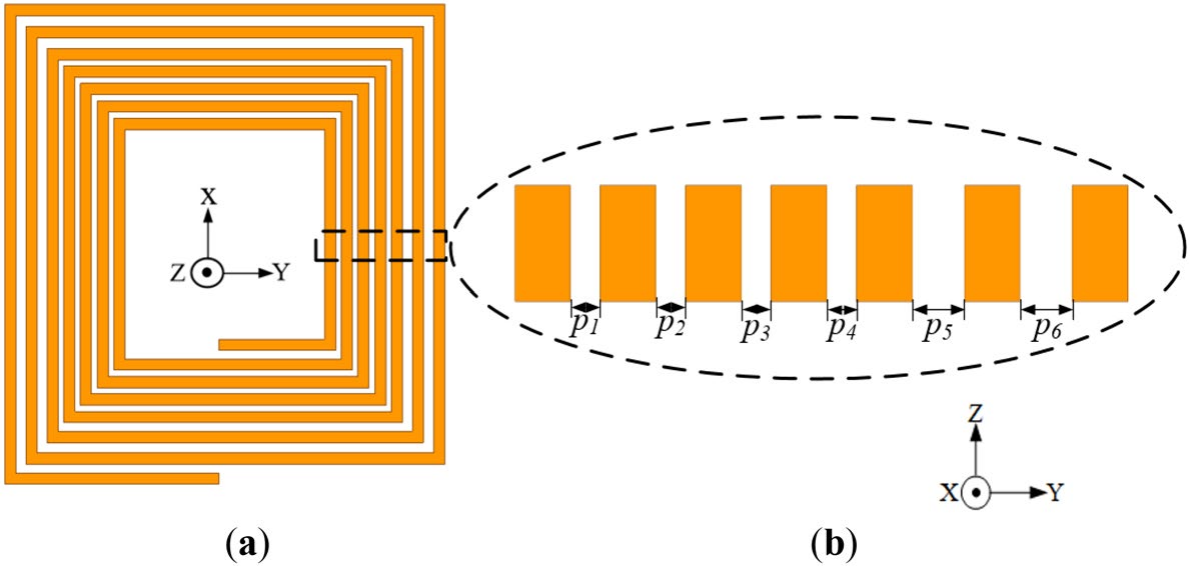
Configuration of the conventional coil structure with wider spacing between outer coil turns relative to the inner turns (reverse structure of Coil-*P*) (**a**) isometric view and (**b**) cross section of the exploded view of each coil turn with different pitches.

Furthermore, a reverse configuration of Coil-*P* where the spacing between coil turns in the outermost section are widened compared to the innermost part of the coil (i.e., *p*_*1*_, *p*_*2*_, *p*_*3*_, *p*_*4*_, *p*_*5*_ and *p*_*6*_ of 0.8, 0.8, 0.8, 0.8, 1.4 and 1.4 mm, respectively) is presented in Fig. 8. Both Coil-*P* and its reverse configuration are simulated at a frequency of 85 kHz.

The simulation results of the *L*, *R* and *Q* for both Coil-*P* and its reverse structure are presented in Table 4. It can be realized that Coil-*P* (i.e., inner coil turns with wider spacing) resulted in a relatively lower resistance of 38.66 mΩ compared to that of the conventional coil with resistance 41.46 mΩ. Also, the reverse structure of Coil-*P* realized a relatively higher resistance of 45.60 mΩ compared to Coil-*P*. In addition, the inductance of Coil-*P* is relatively higher as compared to its reverse configuration, which makes the approach of spacing out the inner neighbouring coil turns a better technique to achieving a higher *Q*.

**Table 4:** Simulation results of the coil with unequal pitches and its reverse structure.

Coil structure (mm)	*L* (μH)	*R* (mΩ)	*Q*
Coil with unequal pitches (*p*_*1*_ = 1.4 , *p*_*2*_ = 1.4, *p*_*3*_ = 1.4, *p*_*4*_ = 1.4, *p*_*5*_ = 0.8, *p*_*6*_ = 0.8)	2.76	38.66	38.13
Reverse structure (*p*_1_ = 0.8, *p*_2_ = 0.8, *p*_3_ = 0.8, *p*_4_ = 0.8, *p*_5_ = 1.4, *p*_6_ = 1.4)	2.57	45.60	30.10

**Figure 9:**
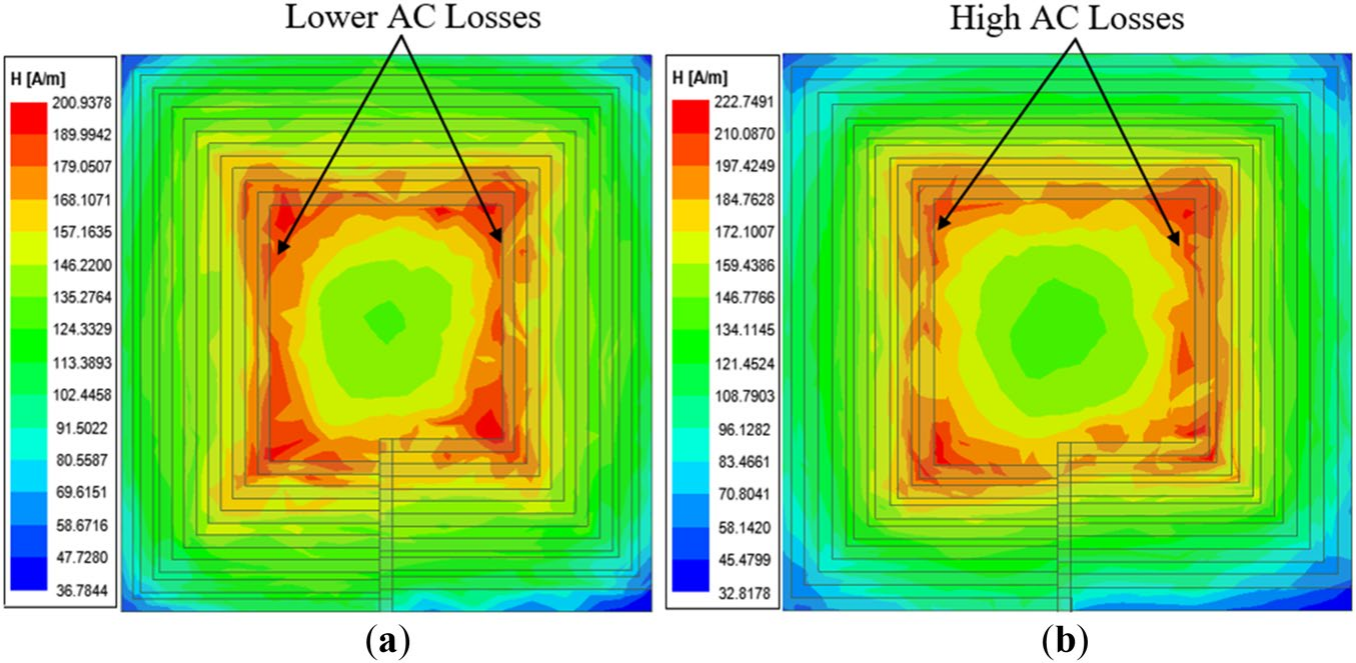
Magnetic field intensity (*H*_*n*_) of rectangular planar coil winding for (**a**) coil structure of wider spacing between inner coil turns (Coil-*P*)^24^, (**b**) coil structure of widened spacing between outer coil turns (reverse structure of Coil-*P*)^24^.

The simulated magnetic field intensity (*H*) results for Coil-*P* and its reverse configuration is given in Fig. 9a,b, respectively. It can be observed that Coil-*P* exhibits lower AC losses compared to the reverse structure which signifies that the former has a reduction in coil resistance as compared to the latter.

### Conventional coil with unequal trace widths

Another method to achieve coil resistance reduction is to employ different widths for each coil turn i.e., employing relatively narrower inner coil turn widths relative to the outer turns. Currents gather at the edges of the conductors when AC currents are fed into the conductors, resulting in increased coil resistance. Thus, the larger the exposed area of the coil, the higher the resistance especially in the inner section of the coil^11,12^.

**Figure 10:**
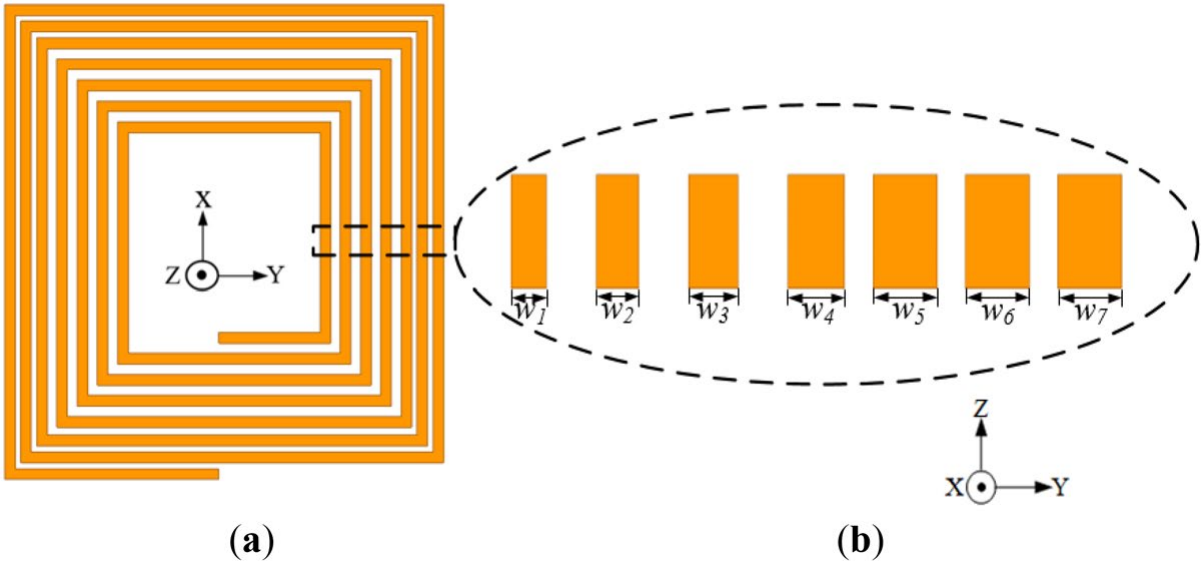
Configuration of the conventional coil structure with narrower inner trace widths relative to the outer turns (Coil-*W*) (**a**) Isometric view and (**b**) cross section of the exploded view of each coil turn with different widths.

The configuration of the conventional coil with trace-width variation (labelled as Coil-*W* for simplicity) is presented in Fig. 10. The coil has constant pitch and turn thickness of 2.4 mm, however with different width for each coil turn (i.e., *w*_*1*_, *w*_*2*_, *w*_*3*_, *w*_*4*_, *w*_*5*_, *w*_*6*_ and *w*_*7*_ of 1, 1.2, 1.4, 1.6, 1.8, 1.8 and 1.8 mm, respectively). Thus, coil turn widths in the inner section where high frequency losses are observed are made narrow leaving less area of the conductor for magnetic field penetration, and this reduces the losses in the inner section of the coil.

**Figure 11:**
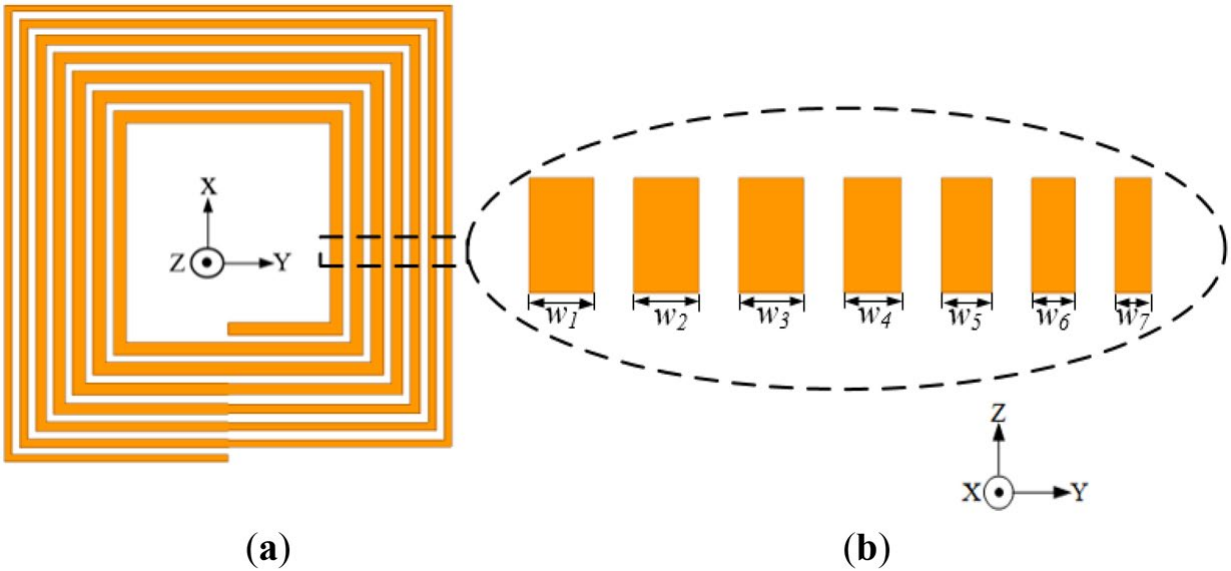
Configuration of the conventional coil structure with wider inner trace width relative to the outer turns (reverse structure of Coil-*W*) (**a**) isometric view and (**b**) cross section of the exploded view of each coil turn with different widths.

The reverse structure of Coil-*W* is presented in Fig. 11, where the inner coil turn widths are relatively wider compared to the outer turns (i.e., *w*_*1*_, *w*_*2*_, *w*_*3*_, *w*_*4*_, *w*_*5*_, *w*_*6*_ and *w*_*7*_ of 1.8, 1.8, 1.8, 1.6, 1.4, 1.2 and 1 mm, respectively). Both coil structures are simulated using the ANSYS Maxwell Electromagnetic software^24^ at a frequency of 85 kHz with the simulation results given in Table 5.

**Table 5:** Simulation results of the coil with unequal coil turn width and its reverse structure.

Coil structure (mm)	*L* (μH)	*R* (mΩ)	*Q*
Coil with unequal coil turn widths (*w*_*1*_ = 1.0, *w*_*2*_ = 1.2, *w*_*3*_ = 1.4, *w*_*4*_ = 1.6, *w*_*5*_ = 1.8, *w*_*6*_ = 1.8,* w*_*7*_ = 1.8)	2.60	36.15	38.41
Reverse structure (*w*_1_ = 1.8, *w*_2_ = 1.8, *w*_3_ = 1.8, *w*_4_ = 1.6, *w*_5_ = 1.4, *w*_6_ = 1.2,* w*_7_ = 1.0)	2.92	46.86	33.28

From the results, it can be realized that Coil-*W* realizes a relatively lower resistance of 36.15 mΩ compared to the reverse structure with 46.86 mΩ. Again, Coil-*W* attains a reduced lower resistance compared to that of the conventional structure, thereby realizing a higher *Q*-factor.

**Figure 12:**
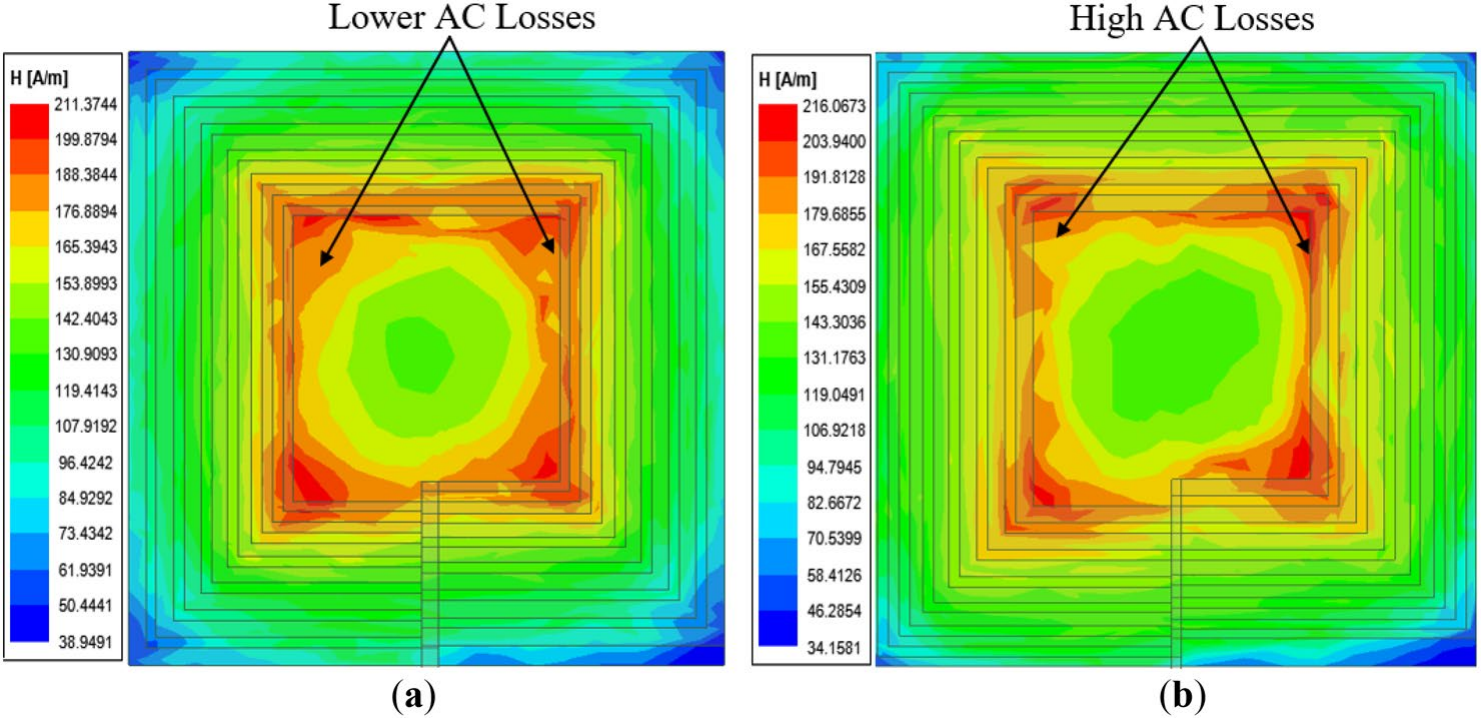
Magnetic field intensity (*H*_n_) of rectangular planar coil winding for (**a**) coil structure with narrower inner coil turn widths (Coil-*W*)^24^ and (**b**) coil structure with wider inner coil turn widths (reverse structure of Coil-*W*)^24^.

The simulated magnetic field intensity results are compared for both Coil-*W* and the reverse and given in Fig. 12. From the results, Coil-*W* exhibits lower AC losses compared to its reverse signifying the achievement of the reduction in the total coil resistance.

To derive the coil width for the individual turns (*W*_*n*_), from the inner turns to the outer turns, a line-width design equation^27^ is utilized as given in (16). Thus, for a spiral inductor with variable coil turn width, *n, N, T, D*_*o*_* and D*_*i*_ represents the number of coil turn from the outermost turn to the innermost coil turns, total number of coil turns, conventional thickness of the coil, outer and inner diameter of the coil, respectively. In addition, *δ* represents the skin-depth of the coil.

16 $$Wn = 1.1\delta \left[ {\frac{{D_{o} - D_{i} }}{(4n - N)T}} \right]^{\frac{2}{3}}$$

### Conventional coil with unequal trace thickness

To improve the *Q* of the coil, the resistance can be reduced by employing varying coil trace thickness where the turns of the inner section of the coil are made shorter relative to the outer turns. This reduces the high AC losses experienced within the centre winding without affecting the coil inductance.

**Figure 13:**
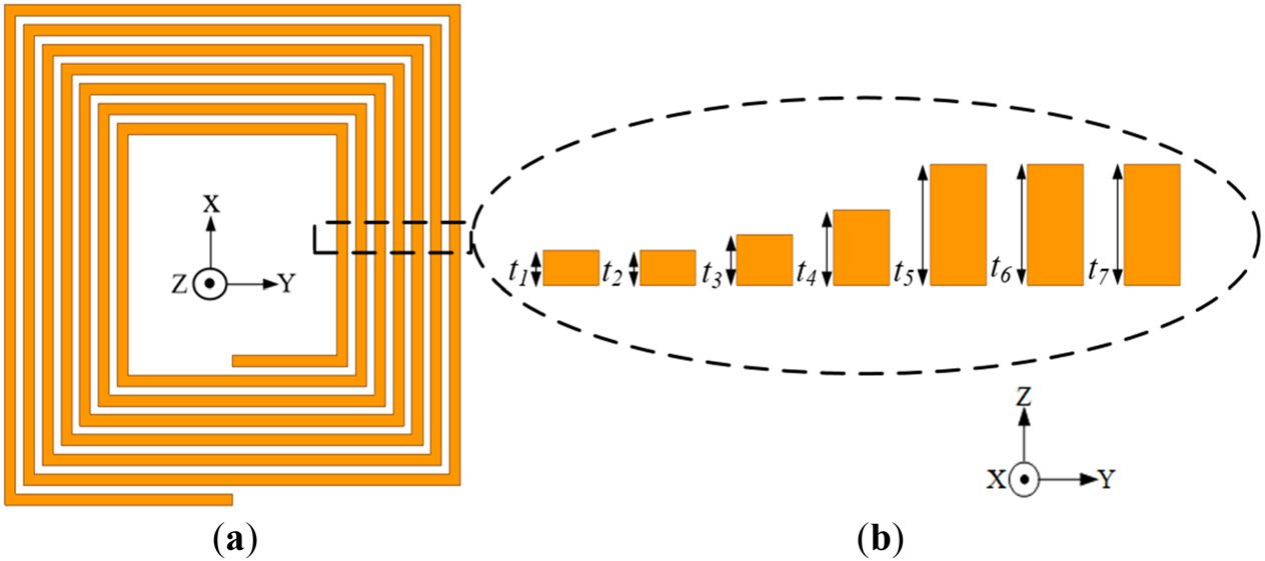
Configuration of the conventional coil structure with reduced inner trace thickness relative to the outer turns (**a**) isometric view and (**b**) cross section of the exploded view of each coil turn with different thickness.

Figure 13 shows the conventional rectangular planar coil incorporated with different trace thicknesses (labelled as Coil-*T*). The coil has thickness i.e., *t*_1_, *t*_2_, *t*_3_, *t*_4_, *t*_5_, *t*_6_, *t*_7_ of 0.7, 0.7, 1, 1.5, 2.4, 2.4, 2.4 mm, respectively from the innermost section towards the outer section of the coil. The coil turn values are selected following a sigmoid-like curve. The coil has a constant width and pitch of 1.5 and 1.1 mm, respectively. Thus, the conductor thickness (*t*) decreases as the coil turns advances to the inner coil section leaving less conductor for high magnetic fields to penetrate, thereby reducing the high-frequency resistance losses.

**Figure 14:**
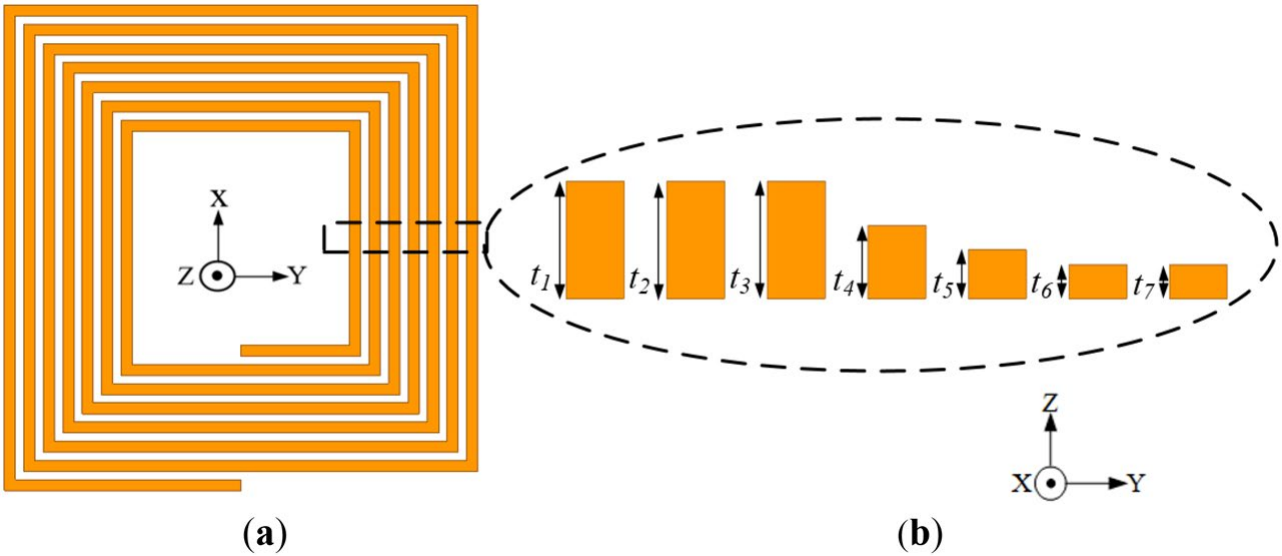
Configuration of the conventional coil with increased inner thickness relative to the outer turns (reverse structure of Coil-*T*) (**a**) isometric view and (**b**) cross section of the exploded view of each coil turn with different thickness.

To verify the turn thickness variation technique, a reverse configuration of Coil-*T* with turn thickness (*t*_1_, *t*_2_, *t*_3_, *t*_4_, *t*_5_, *t*_6_, *t*_7_ of 2.4, 2.4, 2.4, 1.5, 1, 0.7, 0.7 mm, respectively) is designed and presented in Fig. 14. Both Coil-*T* and its reverse structure are simulated at 85 kHz. The EM simulation results are given in Table 6.

**Table 6:** Simulation results of the coil with coil trace thickness variation and its reverse structure.

Coil structure (mm)	*L* (μH)	*R* (mΩ)	*Q*
Coil with trace thickness variation (*t*_*1*_ = 0.7, *t*_*2*_ = 0.7, *t*_*3*_ = 1, *t*_*4*_ = 1.5, *t*_*5*_ = 2.4, *t*_*6*_ = 2.4, *t*_*7*_ = 2.4)	2.95	40.15	39.24
Reverse structure (*t*_1_ = 2.4, *t*_2_ = 2.4, *t*_3_ = 2.4, *t*_4_ = 1.5, *t*_5_ = 1, *t*_6_ = 0.7, *t*_7_ = 0.7)	2.86	46.25	33.02

From the simulation results, Coil-*T* attains a relatively lower resistance of 40.15 mΩ compared to that of the reverse structure with 46.25 mΩ. Comparing the coil resistance of Coil-*T* and the conventional rectangular planar coil structure with 41.46 mΩ, Coil-*T* has a slight reduction. However, Coil-*T* exhibits a higher inductance of 2.95 μH compared to the conventional coil structure with 2.82 μH. Thus, Coil-*T* achieves a relatively higher *Q*-factor than the conventional coil structure.

**Figure 15:**
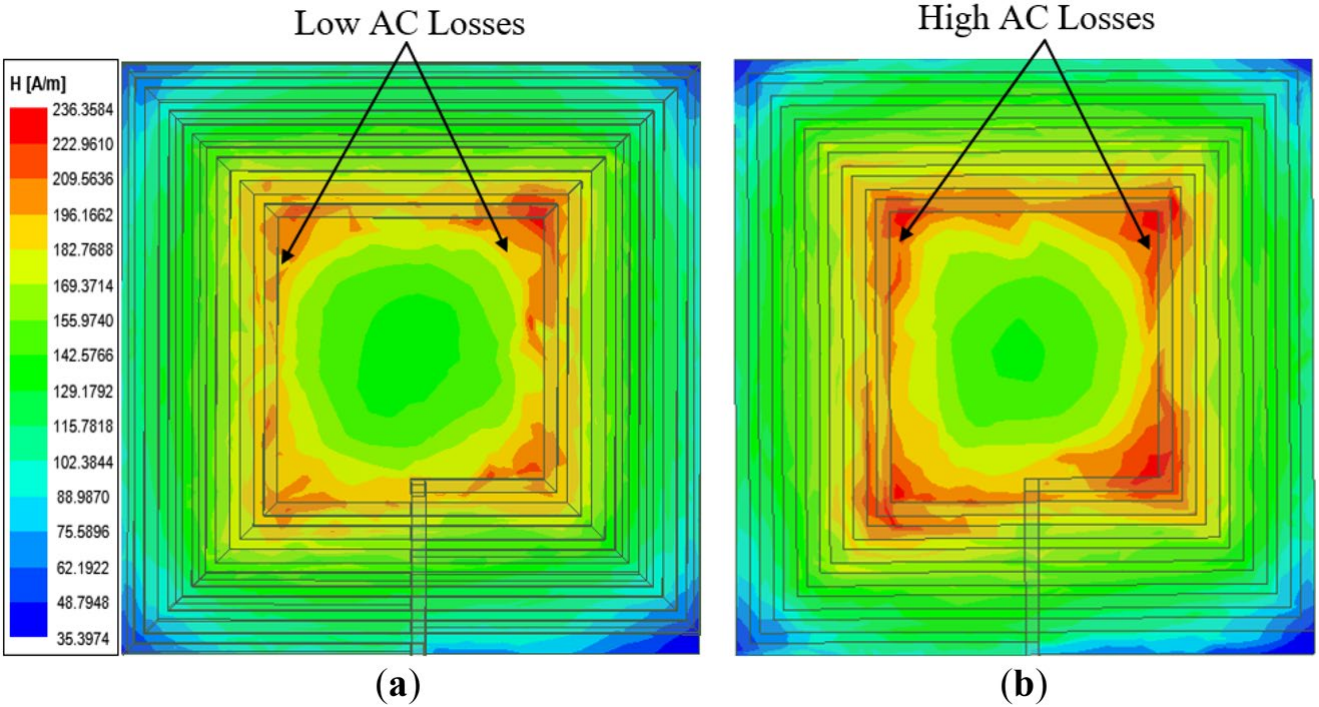
Magnetic field intensity (*H*_*n*_) of rectangular planar coil winding for (**a**) coil structure with reduced inner turn thicknesses (Coil-*T*)^24^ and (**b**) coil structure with increased turn thicknesses (reverse of Coil-*T*)^24^.

Again, from the simulated magnetic field intensity results given in Fig. 15, it can be deduced that Coil-*T* has lower AC losses compared to the reverse structure which illustrates a reduction of the total coil resistance.

### Proposed coil structure

The proposed coil layout harnesses the advantages of the aforementioned techniques to reduce the resistance without compromising on the inductance of the rectangular planar coil. The technique of the trace thickness, width and pitch variation is employed in the proposed coil layout. Therefore, the proposed coil consists of wider spacing in the innermost section relative to the outer part of the coil, narrow inner coil turns relative to the outer turns, and reduced inner coil turn thickness relative to the outer turns.

**Figure 16:**
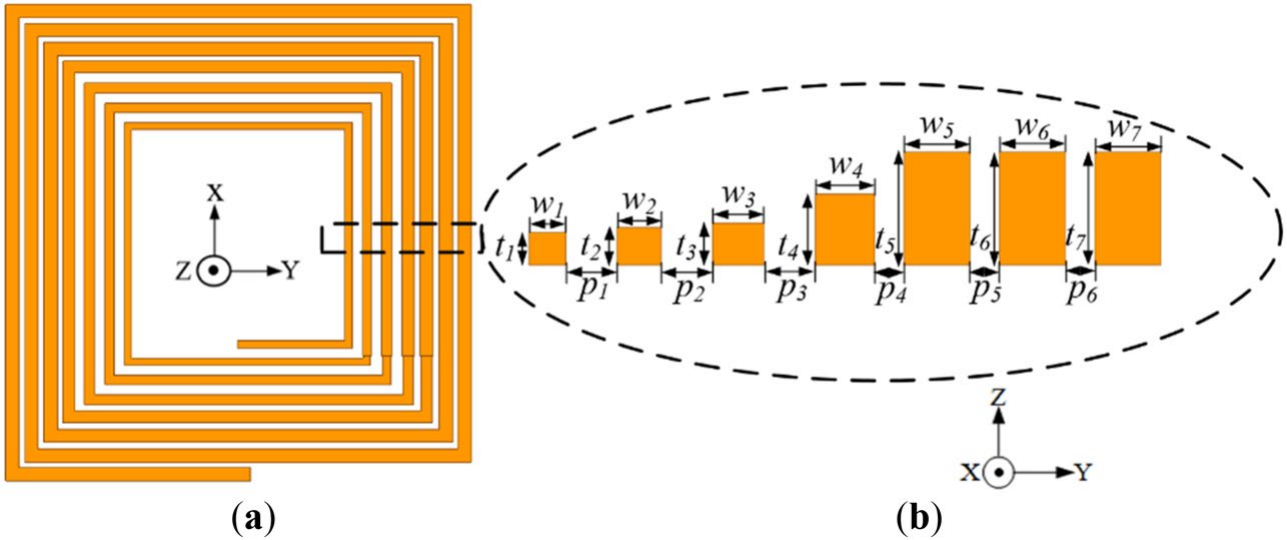
Geometry with coil trace thickness, width, and pitch variation (**a**) isometric view and (**b**) cross section of the exploded view of each coil turn with different thickness and pitch.

The proposed coil is illustrated in Fig. 16 where optimized values of the neighbouring turn spacing (i.e., *p*_*1*_, *p*_*2*_, *p*_*3*_, *p*_*4*_, *p*_*5*_ and *p*_*6*_ of 1.4, 1.4, 1.4, 1.4, 0.8 and 0.8 mm, respectively) and trace thicknesses (i.e., *t*_*1*_, *t*_2_, *t*_3_, *t*_4_, *t*_5_, *t*_6_, and *t*_7_ of 0.7, 0.8, 0.9, 1.5, 2.4, 2.4, 2.4 mm, respectively). High performance WPT coils with miniaturized sizes are mostly desired for WPT applications. In order to maintain the original size of the coil, the inner trace widths are designed to be narrow in order to vary the pitch between neighbouring inner turns, i.e.,* w*_1_, *w*_2_, *w*_3_, *w*_4_, *w*_5_, *w*_6_, *w*_7_ of 1, 1.2, 1.4, 1.6, 1.8, 1.8, 1.8 mm from the inner coil turns towards the outer turns, respectively.

The proposed rectangular planar coil with trace pitch, width and thickness variation reduces the high AC losses in the innermost section of the coil without compromising on the inductance of the coil thereby enhancing the *Q*.

## Experimental results and verification

To verify the proposed *Q*-factor optimization technique, the conventional rectangular planar coil presented in "Conventional coil" section and the proposed planar coil with varying coil thickness (*t*), width (*w*) and pitch (*p*) are simulated in ANSYS Maxwell Electromagnetics software^24^. Both the conventional and proposed coil have the same size (i.e., 6.3 × 6.6 cm^2^).

**Figure 17:**
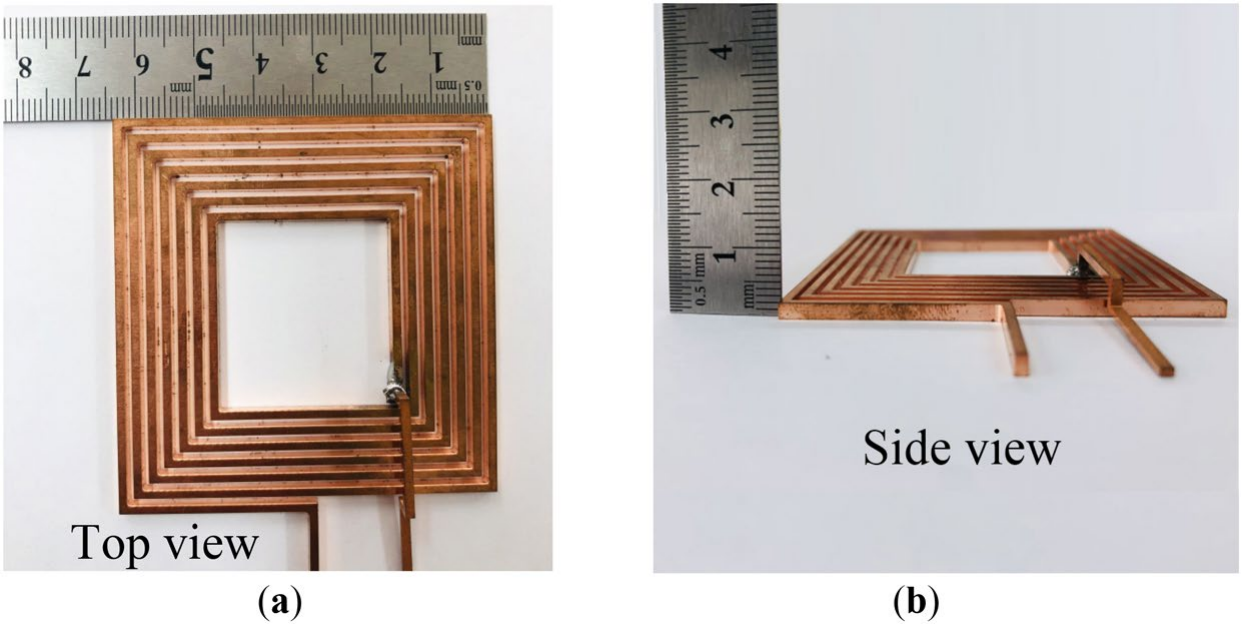
Photograph of the fabricated conventional coil (t = 2.4 mm) (**a**) isometric view and (**b**) side view.

**Figure 18:**
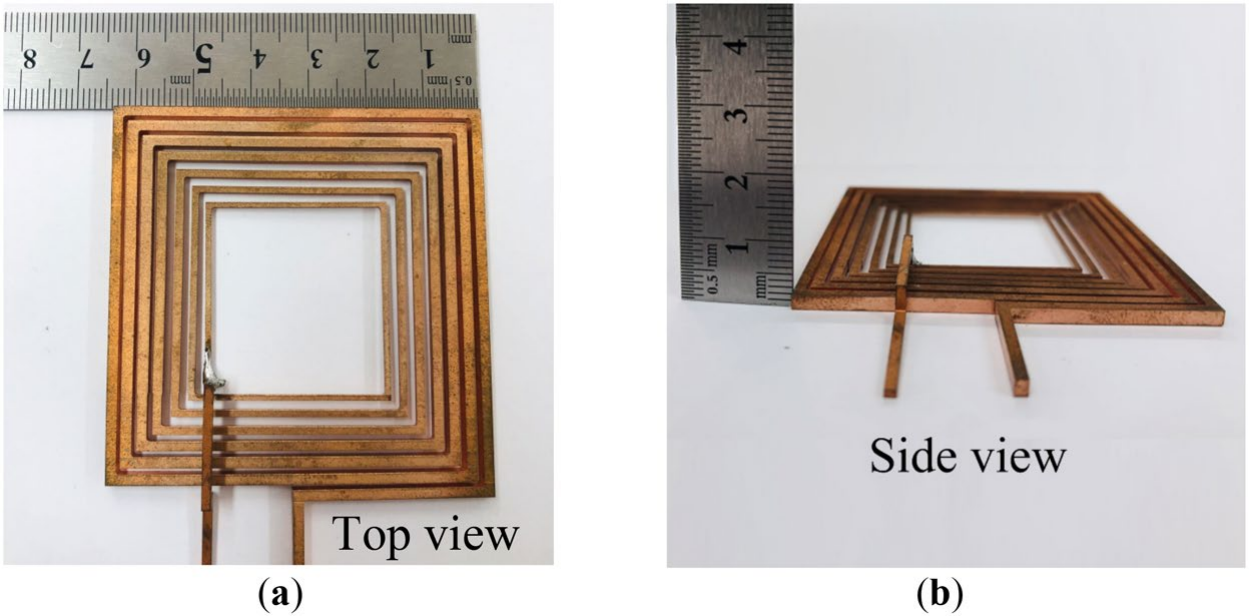
Photographs of the proposed coil structure (**a**) isometric view and (**b**) side view.

To validate the feasibility and efficacy of the proposed coil layout structure, prototypes of the conventional and proposed coil structures are fabricated as shown in Figs. 17 and 18, respectively. All the turns of the conventional coil have the same thickness, width, and pitch between neighbouring coil turns from the innermost part of the coil to its outermost section.

**Figure 19:**
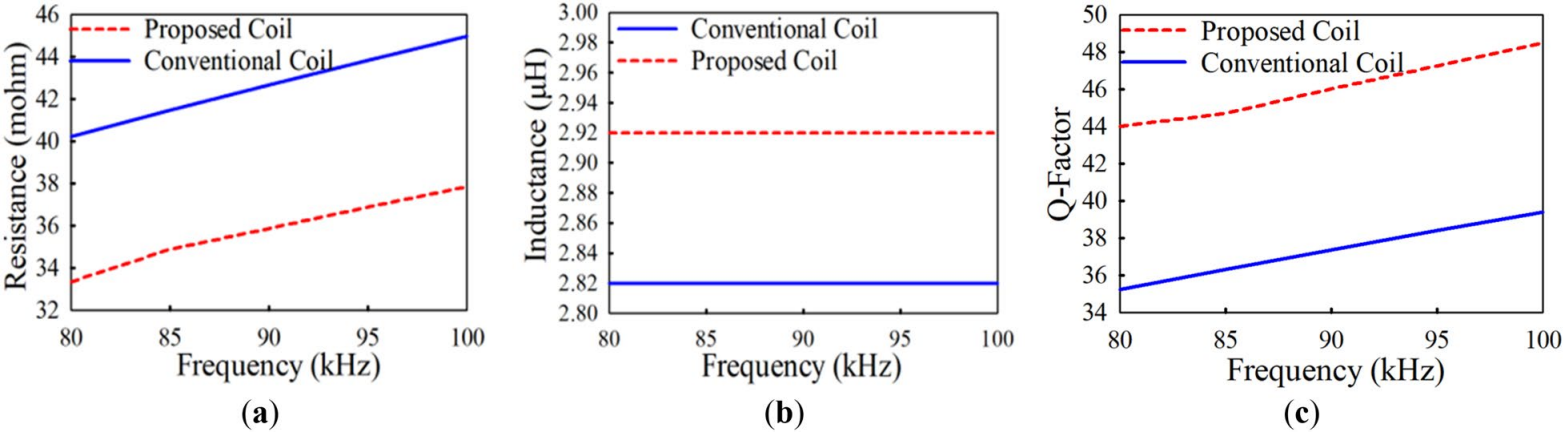
Comparison of the conventional and proposed coil versus frequency in term of (**a**) resistance (**b**) inductance and (**c**) *Q*-factor.

**Figure 20:**
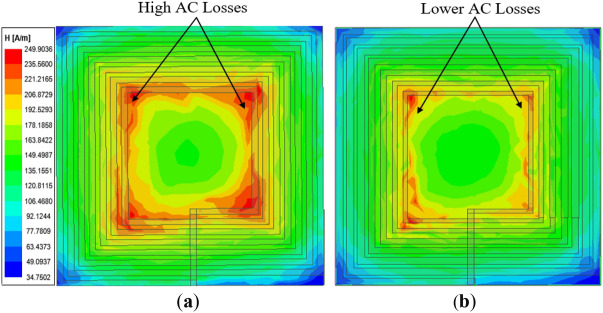
Magnetic field intensity (*H*_*n*_) of rectangular planar coil winding for (**a**) conventional coil^24^ and (**b**) proposed coil structure^24^.

**Figure 21:**
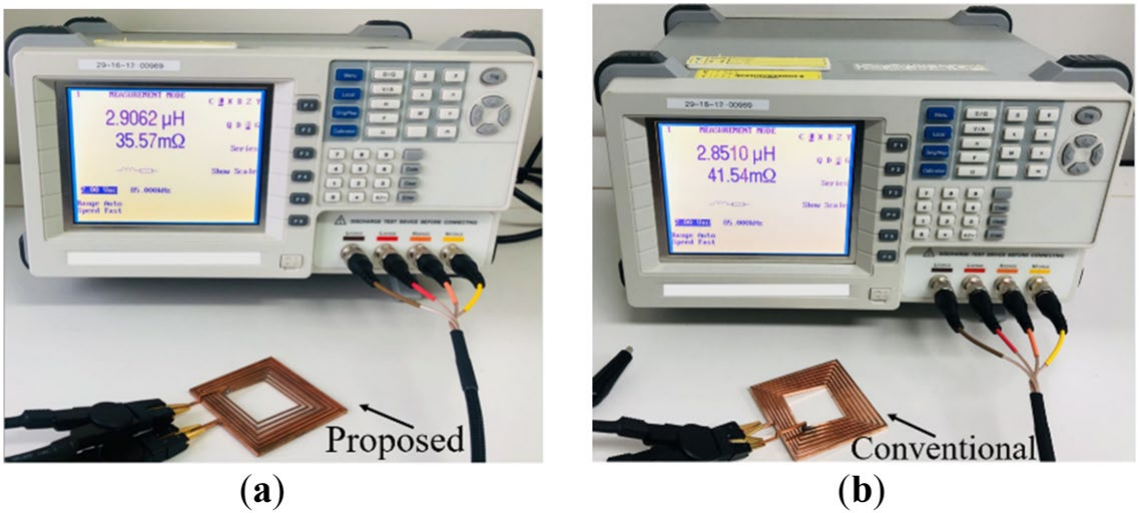
Measurement set-up for fabricated coils for (**a**) proposed coil structure and (**b**) conventional coil structure.

The simulated and measured results for different operating frequencies ranging from 80 to 100 kHz of the inductance (*L*), resistance (*R*) and quality factor (*Q*) for both coils are summarized in Table 7. An LCR meter is used to measure the inductance and resistance of all fabricated coils. A graph to compare the resistance (*R*), inductance (*L*) and quality factor (*Q*) of both the conventional and proposed coil structures is presented in Fig. 19. It can be realized from Fig. 19a and Table 7 that for all the operating frequencies, the proposed coil exhibits lower resistance compared to the conventional coil, for both the measured and simulation results. In addition, it can be noticed from Fig. 19b that the proposed coil realizes a higher inductance compared to the conventional coil. Moreover, the *Q*-factor of the proposed coil is shown to be higher than the conventional coil for all the operating frequencies as shown in Fig. 19c. However, it can be observed that the higher the operating frequency, the higher the *Q*-factor difference between the proposed and conventional coil. Thus, with the implementation of the proposed *Q*-factor optimization technique, the conductor (coil turns) sizes are minimal for high magnetic fields to penetrate, hence lessening the high-frequency resistance losses. This can be deduced from the *H*-field intensity results between the conventional and proposed coil depicted in Fig. 20. It is again evident from the measurement setup of both the conventional and proposed coil at a frequency of 85 kHz as shown in Fig. 21a,b.

**Table 7:** Simulation and measured results comparison of the conventional and proposed for varying frequencies.

Coil structure	*f* (kHz)	Simulation results		Measured results		Quality factor	
		*L* (μH)	*R* (mΩ)	*L* (μH)	*R* (mΩ)	*Q*_*sim*_	*Q*_*meas*_
Conventional coil	80	2.82	40.22	2.81	41.39	35.24	34.13
	85	2.82	41.46	2.85	41.54	36.32	36.64
	90	2.82	42.66	2.81	43.35	37.38	36.65
	95	2.82	43.83	2.80	44.90	38.40	37.22
	100	2.82	44.97	2.80	45.90	39.40	38.41
Proposed coil	80	2.91	33.84	2.84	34.62	43.22	41.23
	85	2.91	34.88	2.91	35.57	44.56	43.69
	90	2.91	35.89	2.84	36.76	45.85	43.69
	95	2.91	36.87	2.85	37.64	47.11	45.20
	100	2.91	37.83	2.87	38.81	48.33	46.46

This technique subsequently enhances the *Q*-factor of the coil. Moreover, the proposed *Q*-factor optimization technique does not compromise on the inductance (*L*) of the coil as seen from measured results in Table 7. Thus, the proposed technique provides a suitable approach where the coil inductance is insignificantly affected while reducing the resistance, which results in a higher *Q*-factor. The discrepancies between the simulated and measured results looking at Table 7 may be due to soldering of the airbridge of the planar coils. Notwithstanding, the effect of the soldering on the *Q*-factor may be negligible.

In an attempt to investigate the effect of a different dielectric substrate (i.e., FR-4, *ɛ*_*r*_ = 4.4) other than air (*ɛ*_*r*_ = 1.0006) on the inductance (*L*), resistance (*R*) and the *Q*-factor of the coils, both the conventional and proposed coils were simulated and compared for varying frequencies as given in Table 8. It can be observed from Table 8 that the *Q*-factor (*Q*) results for both the conventional and proposed coils when the FR-4 dielectric substrate is implemented shows almost same results compared to when the air is utilized.

**Table 8:** Simulation results comparison of the conventional and proposed for different dielectric substrates.

Coil structure	*f* (kHz)	Inductance (μH)		Resistance (mΩ)		Quality factor	
		*L*_*Air*_	*L*_*FR-4*_	*R*_*Air*_	*R*_*FR-4*_	*Q*_*Air*_	*Q*_*FR-4*_
Conventional coil	80	2.816258	2.816915	40.223647	40.141429	35.24	35.27
	85	2.816258	2.816915	41.461586	41.376838	36.32	36.36
	90	2.816258	2.816915	42.663620	42.576415	37.38	37.41
	95	2.816258	2.816915	43.832703	43.743108	38.40	38.44
	100	2.816258	2.816915	44.971404	44.879482	39.40	39.44
Proposed coil	80	2.919761	2.919980	33.843341	33.830469	43.22	43.39
	85	2.919761	2.919980	34.884917	34.871649	44.56	44.72
	90	2.919761	2.919980	35.896284	35.882631	45.85	46.02
	95	2.919761	2.919980	36.879926	36.865899	47.11	47.28
	100	2.919761	2.919980	37.838005	37.823614	48.33	48.51

The proposed coil structure implemented with the *Q*-factor optimization technique is compared to existing coil structures in Table 9. Although parameters such as the coil size, coil shape and operating frequencies are different, this paper compares the efficiency in terms of the *Q*-factor improvement. It is worth noting that, the *Q*-factor enhancement is dependent on the frequency of operation (i.e., from Eq. (14), the higher the frequency, the higher the *Q*-factor enhancement). Thus, the *Q*-factor enhancement tends to increase for higher frequencies. Employing the same frequencies as that of^10,12,13,16,28^, our proposed work resulted in a *Q*-factor enhancement of 23.49%, 23.49%, 23.48%, 23.50% and 23.51%, respectively which shows that the proposed *Q*-factor optimization technique implemented in this work resulted in relatively high *Q*-factor compared to other enhancement methods.

**Table 9:** Comparison of the proposed coil with previous works.

Ref	Coil structure	Frequency	Enhancement method	*Q*-factor increment (%)
^10^	Square	10 MHz	Divide and merge structure	21
^12^	Square	10 MHz	Varying of trace width and turn to-turn spacing	17
^13^	Circular	200 kHz	Non-unity track-width ratio	10
^16^	Square	6.78 MHz	Non-uniform wire width	20.64
^28^	Circular	1 MHz	Track width ratio application	18
This work	Rectangular	85 kHz	Trace width, pitch, and thickness variation	19.24

Moreover, comparing the structures in^10^, the division and merging technique employed is complex in its implementation and has the limitation of being applicable to spiral winding with just a single coil turn. Furthermore, the proposed coil structure has a relatively smaller size compared to the other reference coil structures.

## Conclusion

In this paper, a novel method of enhancing the *Q*-factor of a planar spiral coil, which involves the variation of trace thickness, width, and pitch, has been presented. The method targets the innermost coil turns where high AC losses are concentrated. Narrowing, spacing out and reducing the inner coil trace width, pitch, and thickness, respectively resulted in a reduction of the coil resistance thereby increasing the *Q*-factor of the coil. The proposed *Q*-factor optimization technique can be utilized to design high performance WPT coils, without altering the coil inductance, thereby maintaining the original coil size. The proposed coil achieved a *Q*-factor enhancement of about 19.24% at 85 kHz.

## Data Availability

The datasets used and/or analysed during the current study are available from the corresponding author on reasonable request.
